# Redox Modulations, Antioxidants, and Neuropsychiatric Disorders

**DOI:** 10.1155/2016/4729192

**Published:** 2015-11-10

**Authors:** Erik A. Fraunberger, Gustavo Scola, Victoria L. M. Laliberté, Angela Duong, Ana C. Andreazza

**Affiliations:** ^1^Centre for Addiction and Mental Health, 250 College Street, Toronto, ON, Canada M5T 1R8; ^2^Department of Pharmacology, University of Toronto, Medical Science Building, 1 King's College Circle, Toronto, ON, Canada M5S 1A8; ^3^Department of Psychiatry, University of Toronto, 250 College Street, Toronto, ON, Canada M5T 1R8

## Abstract

Although antioxidants, redox modulations, and neuropsychiatric disorders have been widely studied for many years, the field would benefit from an integrative and corroborative review. Our primary objective is to delineate the biological significance of compounds that modulate our redox status (i.e., reactive species and antioxidants) as well as outline their current role in brain health and the impact of redox modulations on the severity of illnesses. Therefore, this review will not enter into the debate regarding the perceived medical legitimacy of antioxidants but rather seek to clarify their abilities and limitations. With this in mind, antioxidants may be interpreted as natural products with significant pharmacological actions in the body. A renewed understanding of these often overlooked compounds will allow us to critically appraise the current literature and provide an informed, novel perspective on an important healthcare issue. In this review, we will introduce the complex topics of redox modulations and their role in the development of select neuropsychiatric disorders.

## 1. What Are Redox Modulations?

As a dynamic environment, a variety of chemical reactions are constantly occurring within our cells at all times. A common type of reaction, the reduction-oxidation (redox) reaction, plays a vital role in maintaining cellular functions (Table 1) including metabolic cycles (e.g., NAD^+^ and NADH recycling) and detoxification of harmful substances [1]. In these reactions, usually facilitated by an enzyme, one reactant loses electrons (becomes oxidized) and another gains those same electrons (becomes reduced) [1, 2]. As a result, our cells must maintain a delicate electrical balance between the various macromolecules that comprise them. This balance between oxidized and reduced compounds within the cell is known as the redox status [1, 2]. In a healthy cell, this balance is maintained as a result of our natural, endogenous antioxidant defences counteracting the continuous production of reactive species. Under normal conditions, reactive species are commonly produced as by-products of metabolism [3]. Over time, however, our bodies have evolved adaptations to not only detoxify these reactive species but use them to fulfill useful biological functions [4] (Table 1). In cases where this balance of antioxidants and reactive species is disrupted by an* excess* or* deficiency* of either one, our body experiences a strong modulation of its redox status, commonly referred to as oxidative stress [5].

Redox modulation is defined as an imbalance in the redox status. If this imbalance is a shift towards a drastically more oxidized environment, it is characterized by alterations in cellular dynamics and varying degrees of DNA, RNA, protein, and lipid damage [6]. While there are many compounds such as reactive carbon and bromine species that can cause damage to our cells, the focus of this review will be on reactive oxygen species (ROS) and reactive nitrogen species (RNS) due to their high prevalence within our body and the surrounding environment [7].

**Figure 1:**
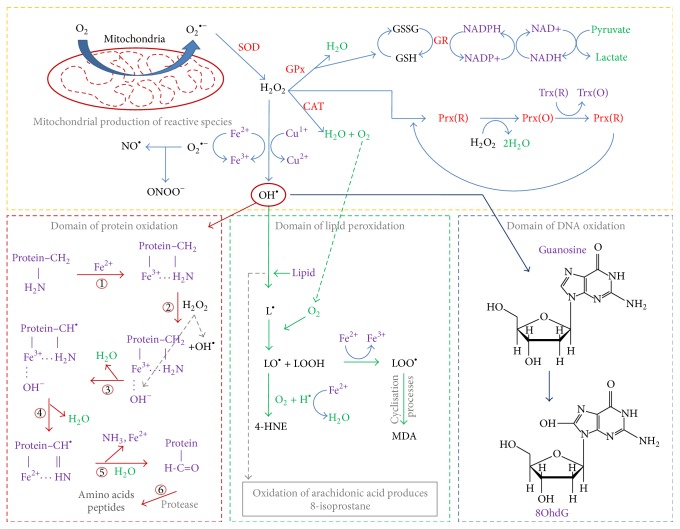
Production of reactive species and the endogenous antioxidant system. Red: enzymes; green: other products; purple: cofactor/substrate; black: reactive species.

As shown in Figure 1, the majority of ROS and RNS species originate from the metabolism of oxygen in the mitochondria [8]. The primary reactive by-product, the superoxide anion (O_2_^•−^), is exported from the mitochondria into the cytosol, via an anion channel, where it proceeds through numerous chemical reactions in our body's attempt to reduce its toxicity. Unfortunately, at the same time and under the correct environmental conditions, the superoxide anion can be converted into additional reactive species either directly or indirectly through catalysis [9]. A common example within the human body is the reduction of hydrogen peroxide into hydroxyl radicals via transition metals, usually iron (Fenton and Haber-Weiss reactions) [10] (following equations):

(1) Fe3++O•2−⟶Fe2++O2Reduction  of  ferric  iron

(2) Fe2++H2O2⟶Fe3++OH−+OH•Fenton  Reaction

(3) O•2−+H2O2⟶OH•+OH−+O2Net  Reaction

## 2. Macromolecular Changes Caused by Reactive Species

**Figure 2:**
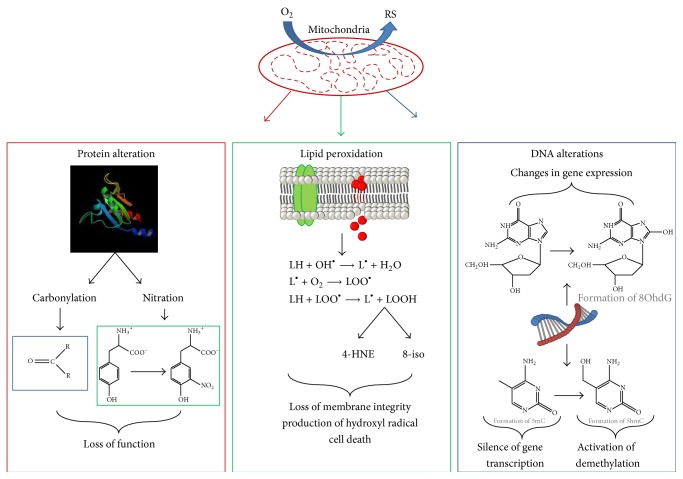
Examples of the effects of reactive species in the cell.

Once these toxic molecules are produced in the body, they begin to interact with DNA, lipids, and proteins to cause damage, leading to an alteration in cellular function (Figure 2). It is important to remember that although the effects shown in Figure 2 are negative, not all changes caused by reactive species are detrimental to the body [4]. In fact, recent evidence has provided support for the hypothesis that posttranslational modifications, such as carbonylation, S-nitrosylation, and nitration, play a vital role in the degradation of unnecessary or damaged proteins, maintaining cellular health [11, 12]. A second example is the regulation of cellular development by H_2_O_2_, considered to be a key component in mediating the cell cycle and the aging process [13]. At different concentrations, hydrogen peroxide influences the cell to advance or halt the cell cycle. For example, at* in vivo* concentrations of 10^−8^, 10^−6^, and 10^−4^ M, H_2_O_2_ causes the cell to proliferate, cease its growth, or initiate apoptosis, respectively [13].

An important issue to address is the point at which the oxidative damage that is initially beneficial becomes harmful to the cell. In order to differentiate between negative and positive effects of reactive species on the body, we must analyze several determining factors including the concentration, half-life, and diffusibility of the reactive species produced. When the cell is utilizing these molecules for signalling purposes, they usually possess very short half-lives and very limited diffusibility or are present in low concentrations [14]. For example, monocytes and neutrophils use NADPH oxidase to produce the superoxide anion as a defence against bacterial or fungal infection [15]. Considering that the superoxide anion has a very short half-life (10^−6^ s), a very limited ability to diffuse throughout the cell, and is generated in a small concentration onto a focused target (i.e., bacteria or fungi) [14], it is generally viewed as beneficial in this context. Numerous other examples, indeed derivatives from reactions involving the superoxide anion, including H_2_O_2_ and hypochlorite, also play an important role in the neutralization of harmful pathogens and maintenance of a healthy cell. Similarly, antioxidants also play an important role in maintaining cellular functions in the face of redox modulations through a variety of mechanisms.

## 3. What Is an Antioxidant?

As the name implies, antioxidants are compounds that neutralize reactive species by decreasing their reactivity in the body [2]. We can divide antioxidants into two broad categories: endogenous and exogenous. The antioxidants within the body are composed of antioxidant enzyme defenses (Table 2) and additional antioxidant compounds such as melatonin and glutathione that are internally synthesized.

Outside of the body, antioxidants can be supplied by the diet with a wide variety of natural and synthetic compounds found in complex mixtures (such as chocolate or olive oil) or isolated to be taken as a supplement [16]. The mechanism of action of each antioxidant will vary depending upon location, chemical structure, and bioavailability within the body as well as the degree of redox modulation experienced by the cell.

## 4. The Endogenous Antioxidant Response System

**Figure 3:**
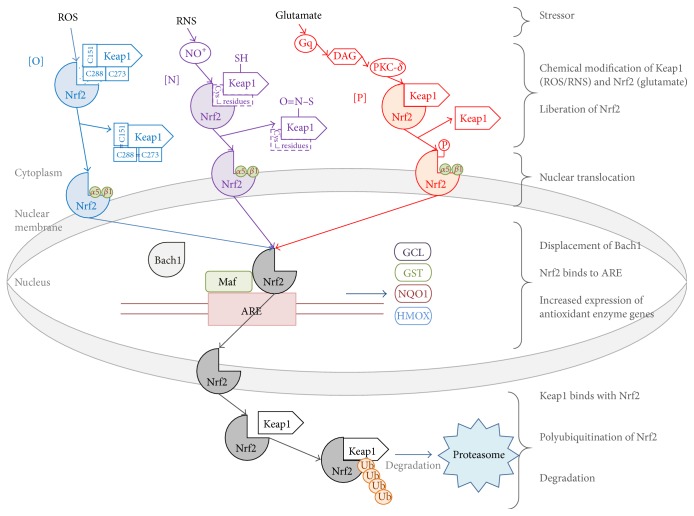
Mechanisms of Nrf2 activation and degradation. ROS, reactive oxygen species; RNS, reactive nitrogen species; Nrf2, nuclear factor- (erythroid-derived-2-) like 2; Keap1, Kelch-like ECH-associated protein 1; DAG, diacylglycerol; PKC, protein kinase C; Bach1, transcription regulator protein BACH1; Maf, transcription factor Maf; ARE, antioxidant response elements; GCL, glutamate-cysteine ligase; GST, glutathione-S-transferase; NQO1, NADPH:quinone oxidoreductase 1; HMOX, heme oxygenase; Ub, ubiquitin.

Under conditions of oxidative/nitrosative stress, the antioxidant response system (ARS) becomes active in order to ensure cellular survival and restoration of a balanced redox status [17]. In our body, Nrf2 acts as a master control for most of our antioxidant defenses, including the ones in the brain. As shown in Figure 3, a stressor can act directly or indirectly to influence the activation of the Nrf2 signal transduction pathway. In fact, Habas et al. report that neuronal activity at the tripartite synapse regulates Nrf2 activity in astrocytes [18]. Following an increase in neuronal activity signalled through neurotransmitters such as glutamate, the astrocytic Nrf2 signalling cascade is triggered via stimulation of group I metabotropic glutamate receptors and Ca^2+^_i_. Regardless of the stressor in question, the translocation of Nrf2 into the nucleus can be accomplished in two primary ways: chemical modification of cysteine residues on Keap1 and/or phosphorylation of Nrf2 [19].

In the kinase-independent mechanism of Nrf2 dissociation, reactive species directly oxidize (C151, C273, and C288) [20] or nitrosylate [21] key cysteine residues on Keap1, a protein bound to Nrf2 that facilitates its polyubiquitination and subsequent degradation under normal conditions. This process creates chemically modified cysteine residues (oxidized disulphide bridges or S-nitrosothiol groups) that allow for Nrf2 to become free within the cytosol.

In the kinase-dependent mechanism of Nrf2 dissociation, a stressor, such as glutamate, activates the G_q_ pathway, leading to the phospholipase C (PLC) catalyzed breakdown of phosphatidylinositol 4,5-bisphosphate (PIP_2_) into diacylglycerol (DAG) and inositol 1,4,5-triphosphate (IP_3_) [22]. The membrane bound DAG acts as a physiological activator of PKC which proceeds to subsequently phosphorylate S40 on Nrf2 [23]. According to recent studies, although PKC-*β* is the most abundant isoform in astrocytes [24], the predominant PKC isoform that participates in the phosphorylation of Nrf2 is PKC-*δ* [25]. Since the delta isoform of PKC is novel, it only requires DAG alone to become active [25] and, as such, the mechanism of increased [26] Ca^2+^_i_ by IP_3_ is discussed elsewhere [22].

As a result of one or both of the mechanisms above, numerous importins, including *α*5 and *β*1, bind to the newly exposed NLS on Nrf2 to facilitate nuclear translocation [27]. Once inside the nucleus, Nrf2 displaces Bach1, a transcriptional repressor of antioxidant response elements (ARE), and heterodimerizes with transcription factor Maf to bind to ARE on the DNA [28]. Consequently, increased expression of endogenous antioxidant enzyme genes such as* NQO1*,* HMOX-1*,* GCL*, and* GST* occurs, increasing cellular defenses against detrimental redox modulations [29].

Once the cell has effectively compensated for the redox modulation, a deactivation cascade commences involving the phosphorylation of glycogen synthase kinase 3*β* (GSK3*β*) via unknown tyrosine kinases [28]. GSK3*β* proceeds to phosphorylate Fyn, a nonreceptor protein-tyrosine kinase, to facilitate its translocation into the nucleus. Once inside the nucleus, Fyn phosphorylates Y568 on Nrf2 to facilitate nuclear export which is immediately followed by Keap1 association, polyubiquitination, and proteolysis.

Considering that our endogenous antioxidant response system is able to tightly regulate the amount of reactive species and minimize related cellular damage, the role of exogenous antioxidants seems, on the surface, superfluous. However, Kaspar et al. [28] found that exogenous antioxidants have a priming effect on the antioxidant response system [19]. Following approximately 0.5–1 hour after exposure, antioxidants were found to induce the phosphorylation of Keap1 (Y85), Fyn (Y213), and Bach1 (Y486) via unknown tyrosine kinases to facilitate their export out of the nucleus. The overall effect of nuclear exportation of negative regulators of Nrf2 is reduced competition for ARE (with Bach1) and decreased nuclear export and degradation of Nrf2 via Fyn and Keap1. Working together with our endogenous antioxidant response system, exogenous antioxidants allow for a more enhanced and efficient defense against detrimental redox modulations.

## 5. Mechanisms of Action of Exogenous Antioxidants

**Figure 4:**
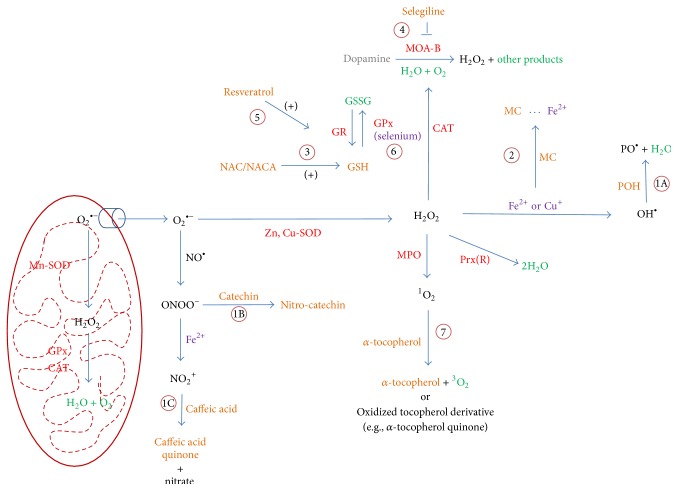
Mechanisms of action of exogenous antioxidants. Red: enzymes; green: other products; purple: cofactor/substrate; black: reactive species; CAT, catalase; MC, metal chelator; POH, polyphenol; GSSG, oxidized glutathione; MPO, myeloperoxidase. Reaction legend 1A: H-atom transfer, 1B: electron donation, 1C: direct scavenging, 2: metal chelation, 3: restoration of endogenous antioxidants, 4: inhibition of RS generating species and reactions, 5: support of endogenous antioxidant enzymes, 6: cofactor in antioxidant enzymes, 7: singlet oxygen quenching.

Aside from enhancing the efficiency of antioxidant gene regulation, exogenous antioxidants also exert their effects through additional mechanisms of action. In cases such as tocopherols and resveratrol, 2 or 3 different actions can be simultaneously carried out to counter the effects of detrimental redox modulations [30, 31]. Shown in Figure 4 below are several examples of antioxidant reactions that take place in the body. In general, there are several common antioxidant mechanisms of action as described in Figure 4.

### 5.1. Hydrogen Atom Transfer, Electron Donation, and Direct Radical Scavenging

In free radical scavenging there are three known primary mechanisms of action: hydrogen atom transfer (Reaction 1A), electron donation (Reaction 1B), and direct radical scavenging (Reaction 1C). In H atom transfer, a reactive hydrogen-containing group on the antioxidant compound undergoes homolytic fission, generating a hydrogen radical and antioxidant radical [32]. The hydrogen radical is then able to interact with the free radical, creating a less reactive species. The antioxidant radical, while still reactive, is relatively less dangerous and can bind with another antioxidant radical to form a nonreactive dimer. In electron donation, the antioxidant compound, containing a conjugated system, donates an electron to the reactive species, producing an anion [32–35]. Using its conjugated system, the antioxidant is able to electronically redistribute the positive charge throughout its chemical structure or adopt an alternative, stable conformation as is the case for catechol containing compounds such as catechins [36] or caffeic acid [37]. In the particular case of caffeic acid, the compound initially undergoes deprotonation under physiological pH conditions, allowing for electron donation to occur from the catechol-like moiety, effectively reducing the nitronium ion [37]. In direct radical scavenging, the antioxidant absorbs a radical into its structure, producing a less reactive final product that possesses reduced cytotoxicity [33, 38, 39].

### 5.2. Metal Chelation

In order to chelate metals, the antioxidant must contain free electron pairs with which to form coordinate or normal covalent bonds with the free metal ion [40]. Common examples of antioxidant ligands include polyphenols [41] and various flavonoids [42]. However, it is also possible to have other antioxidants using sulfur or nitrogen atoms to chelate metal ions with or without a resulting de-protonation [40]. Once the metal ion and antioxidant interact, the antioxidant donates electrons to the metal ion (the number is dependent upon the nature of the covalent bond as described above), reducing it to its ground electronic state and inhibiting its ability to participate in RS generating reactions.

### 5.3. Restoration of Antioxidant Levels

As our body works to maintain the redox status, our endogenous supply of antioxidants begins to diminish, effectively reducing our capacity to fight excessive amounts of reactive species. In order to supplement our antioxidant defenses, we can ingest food or supplements containing natural or synthetic compounds that either get directly converted into the endogenous antioxidant or aid in its replenishment. Two representative examples of lipoic acid and N-acetylcysteine and its amide are explained below.

#### 5.3.1. N-Acetylcysteine (NAC) and N-Acetylcysteine Amide (NACA)

Once NAC or NACA reaches the cells, it is absorbed into the cytosol where it is hydrolyzed to release cysteine, the limiting reagent in the formation of GSH [43]. Using *γ*-glutamylcysteine synthetase, glutamine and cysteine are combined into *γ*-glutamylcysteine where a further addition of glutamine produces glutathione.

#### 5.3.2. Reduced Lipoic Acid

In its reduced form, dihydrolipoic acid (DHLA) can aid in the restoration of endogenous antioxidants including vitamin C, vitamin E, and GSH by acting as a reducing agent [44].

### 5.4. Inhibition of RS Generating Enzymes and Reactions

Commonly used as an adjunct therapy for Parkinson's disease (PD), selegiline acts as a selective irreversible inhibitor of monoamine oxidase B (MAO-B) [45]. By doing so, selegiline increases dopamine availability and reduces the required dosage of L-DOPA, minimizing side effects. A secondary effect of this drug is to reduce the amount of hydrogen peroxide, a natural by-product of dopamine metabolism, in the neuron [45].

### 5.5. Promote Activities of Antioxidant Enzymes

Certain antioxidants play an indirect role in the protection of cells against oxidative stress by modulating the expression of some endogenous antioxidant enzymes. Two examples of such a mechanism of action involve lipoic acid and resveratrol.

#### 5.5.1. Lipoic Acid

This exogenous antioxidant has the ability to alter the expression of phase II metabolic enzyme genes (conjugating enzymes such as UDP-glucosyltransferase, sulfotransferases, and glutathione-S-transferases) through Nrf2 dependent pathway [44].

#### 5.5.2. Resveratrol

Among its many other mechanisms of action, resveratrol has been shown to induce sirtuin activity [46] leading to nuclear translocation of the FOXO transcription factor [47], an increase in FOXO3a transcription and upregulation of mitochondrial Mn-SOD [48].

### 5.6. Cofactor in Antioxidant Enzymes

In order for the endogenous antioxidant enzymes to work properly, they require numerous cofactors from organic (heme and flavin) and inorganic (metal ions) sources. A common enzyme participating in the detoxification of reactive species is cytosolic glutathione peroxidase which requires a selenium cofactor bound to a cysteine residue to act as a catalytic site for the enzyme. The mechanism involves hydroperoxides or peroxynitrites oxidizing the selenol on the selenocysteine active site on GPx to create less reactive alcohols and nitrites, respectively [49]. The oxidized selenocysteine is reduced via two units of GSH into its corresponding selenic acid. It has also been proposed that thioredoxin reductase may reduce oxidized selenocompounds at the expense of NADPH [50].

### 5.7. Singlet Oxygen Quenching

Certain antioxidants, such as the tocopherols (Vitamin E), exhibit a potent quenching effect when reacting with singlet oxygen. The two known methods by which singlet oxygen is neutralized involve physical or chemical quenching of the excited electronic state. While each of these processes is not mutually exclusive in solution (or in our case,* in vivo*), physical quenching is usually the predominant mechanism [30].

In physical quenching, a charge transfer occurs following an electronic interaction between the singlet oxygen and the tocopherol that results in the singlet oxygen molecule being deactivated to its triplet configuration [30]. It is hypothesized by Gorman et al. (1984) that this occurs via intersystem crossing induced by spin-orbit coupling [51]. Chemical quenching has the same net effect as physical quenching, resulting in a deactivation of a singlet oxygen molecule. However this mechanism of quenching involves the incorporation of the singlet oxygen molecule into the tocol structure to create a quinone and/or quinone-epoxide as well as other oxidized products [30].

Through our understanding of antioxidant mechanisms of action, it becomes possible to hypothesize which compound would be best suited to counteract a neuropsychiatric disease. For example, a hallmark of PD pathology is excess iron in the substantia nigra pars compacta that subsequently generates reactive species via the Fenton and Haber-Weiss reactions [52]. Therefore, a possible strategy to combat PD would be to utilize a compound with iron chelation properties [53] such as flavonoids or DHLA. However, although antioxidants possess many positive functions within our body, like any pharmacologically active compound, they also have side effects and in some cases detrimental effects. Some potential problems surrounding antioxidants will be covered in the following section.

## 6. Limitations of Antioxidants

At first glance, antioxidants appear to be a panacea. However, as with any pharmacologically active compound, there are limitations to their usage and effectiveness within the body. These limitations are mostly concerned with the dosage/concentration, route of administration, possible drug interactions, and negative side effects of the antioxidants.

### 6.1. Dosage/Concentration

In order to demonstrate this point effectively, we will examine the case of the amyloid-*β* peptide, one of the major contributing factors in the pathophysiology of Alzheimer's disease (AD) [54]. In a patient that is not exhibiting symptoms of AD, there is a very small concentration (0.1–1.0 nM) of the amyloid-*β* peptide present in the CSF and plasma [55]. At these low physiological concentrations, amyloid-*β* exhibits antioxidant effects using a hydrophilic moiety to chelate transition metals (Cu and Fe ions) as well as a cysteine residue on Met35 as a free radical scavenger to prevent lipoprotein oxidation [55]. In fact, in comparison to the well-known antioxidant ascorbate, amyloid-*β* levels correlate better with oxidative resistance in the CSF [56]. However, at higher physiological (amyloid-*β*), usually in the *μ*M range, and in the presence of transition metals, the peptide demonstrates prooxidant activity [55]. This general principle of toxicity in proportion to the administered dose can be widely applied to almost every pharmaceutical including exogenous antioxidants.

### 6.2. Route of Administration

In the context of antioxidants, the most common method of administration is oral due to its high compliance among patients. Considering that oral intake of antioxidants is most relevant, it is worth noting that first-pass metabolism, dietary intake, and BBB permeability have dramatic effects on the cerebral absorption and bioavailability of the ingested antioxidant [57]. A prominent example would be the antioxidant selegiline. When administered as an adjunct therapy with L-DOPA for the treatment of PD, it is recommended that the patient ingests a high-fat meal to increase the absorption of the drug due to its hydrophobic properties [45].

### 6.3. Drug Interactions

As a consequence of the combination of aging populations and a rise in popularity of nutritional supplements, interactions between antioxidants and pharmaceuticals constitute an emerging area of research and inquiry. From a pharmacodynamics perspective, antioxidants could act as competitive or noncompetitive antagonists (reversible or irreversible) with medication, effectively reducing the therapeutic window of the medication. A prominent example is the possible physiological antagonism of nifedipine, an antihypertensive agent, by melatonin [58]. Melatonin is an endogenous antioxidant that plays an important role in protecting against free radical-induced oxidative damage [59]. While the exact mechanism is unknown, melatonin is thought to interfere with nifedipine's mechanism of action through directly interacting with several enzymes involved in calcium signalling including calmodulin or adenylate cyclase.

From a pharmacokinetic perspective, in contrast to a pharmacodynamic interaction, the antioxidant would affect the concentration of the medication at several sites including the gastrointestinal tract, binding to plasma proteins, metabolism by CYP enzymes, and renal clearance. A popular example involves the inhibitory interactions between components of certain fruits such as grapefruit, known as furanocoumarins, and intestinal CYP3A4 [60]. Upon ingestion of the furanocoumarins, intestinal CYP3A4 is inhibited, leading to increased oral bioavailability of a drug [60]. Considering that the half-life of the drug is unchanged, this can lead to an unsafe peak plasma concentration within the patient. A similar effect can be found following ingestion of curcumin, an antioxidant component of turmeric [61]. In a study by Burgos-Morón et al., curcumin has been shown to inhibit cytochrome P450 enzymes, glutathione-S-transferase, and UDP-glucuronosyltransferase, leading to a potentially toxic increase in the concentration of any medications that a patient may be taking.

### 6.4. Negative Side Effects

One of the most important issues to address in this context is the false equivalency between “natural” and “safe” often made by those who are wary of the side effects and sceptical of the efficacy of modern pharmaceuticals. As mentioned earlier, the dosage of any pharmacologically active compound must be carefully regulated in order to stay within the experimentally determined therapeutic window. Once the given intake exceeds the median toxic dose (TD_50_), negative side effects can begin to manifest themselves as distressing physical symptoms. One example of negative side effects can be seen in the mechanism of action of a key ingredient in green and black teas, epigallocatechin gallate (EGCG) [62]. Purported as a strong antioxidant, EGCG also displays cytotoxicity* in vitro* in both cancerous and primary human cell lines. Whether these effects can be translated into an* in vivo* context remains to be seen.

Overall, these examples highlight the desperate need for more peer-reviewed research into the efficacy and toxicity of antioxidant compounds that are currently being ingested by the public.

## 7. How Are Oxidative Stress and Antioxidants Relevant to Brain Health?

**Figure 5:**
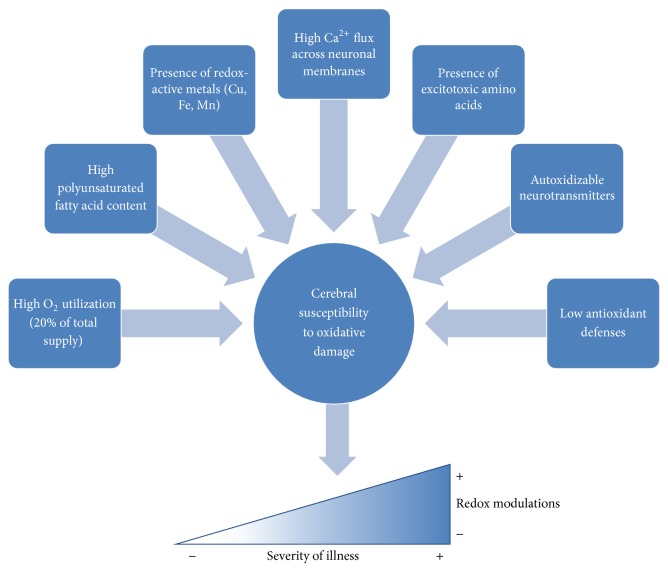
Contributing factors to the cerebral susceptibility to oxidative damage.

Thus far, we have considered oxidative stress in a cellular context. However, considering that the body is much more than the sum of its parts, it is important to apply our mechanistic and cellular understanding of oxidative stress to the general concept of brain health. According to Halliwell and Emerit et al., the brain possesses several key physiological features that make it susceptible to oxidative stress (Figure 5) [7, 63]. (1)* High *O_2_ *Utilization*. Relative to the rest of the body, the brain accounts for a small fraction of body weight. However, since it uses a high supply (up to 20%) of available oxygen, toxic by-products such as hydrogen peroxide and superoxide are inevitably produced and begin to cause damage. (2)* High PUFA Content*. The neuronal membrane consists of numerous polyunsaturated fatty acids (PUFA), notably docosahexaenoic acid (DHA). Vulnerable to oxidation by reactive species, PUFA can be oxidized into radicals and 4-hydroxynonenal (4-HNE), a cytotoxic compound that interferes with neuronal metabolism. (3)* Presence of Redox-Active Metals*. In the average adult brain, there is approximately 60 mg of nonheme iron usually bound to ferritin and hemosiderin. In a normal, healthy brain, the movement of iron into the brain is controlled via transferrin and its associated receptors. However, if there is damage to the brain, especially in areas with high iron content (substantia nigra, caudate nucleus, putamen, and globus pallidus), iron is released from ferritin or diffuses through damaged microvasculature. Once inside the brain, this catalytic iron causes extensive amounts of damage due to the negligible iron-binding ability of the CSF. (4)* High *Ca^2+^ *Flux across Neuronal Membranes*. In the presence of reactive species such as H_2_O_2_, disruptions in mitochondrial and endoplasmic reticulum function, specifically to their calcium sequestration abilities, can cause a rise in intracellular Ca^2+^. This causes the production of reactive species by mitochondria to increase and cause further damage. It has additionally been reported by Fonfria et al. that, in the presence of reactive species such as H_2_O_2_, some neurons and glial cells allow for Ca^2+^ influx via specific cation channels, initiating a detrimental cascade that culminates in cytoskeletal damage [64]. (5)* Excitotoxic Amino Acids*. Once reactive species have induced a state of oxidative stress in neurons, there is a release of glutamate following cell death. This excitatory neurotransmitter proceeds to bind to glutamate receptors on neighbouring neurons, causing cation (Ca^2+^ and Na^+^) influx and eventually necrosis. This initiates an excitotoxic “chain reaction” in which neurons continually experience excessive extracellular glutamate levels. The problem is further compounded by disruptions in glutamate transporters and glutamine synthetase activity. (6)* Autoxidizable Neurotransmitters*. Catecholamine neurotransmitters (dopamine, epinephrine, and norepinephrine) can react with O_2_ to produce superoxide and quinones/semiquinones that readily bind to sulfhydryl side chains and deplete the already low cerebral GSH reserves. (7)* Low Antioxidant Defenses*. Throughout the brain there are lower levels of antioxidant defenses relative to the rest of the body. The only substantial antioxidant enzyme in the brain is catalase, which is very limited in its ability to detoxify H_2_O_2_ since it is localized to microperoxisomes.

As a result, various neuropsychiatric diseases manifest themselves as exploitations of these substrates and cofactors that usually contribute to normal brain health. The main sources of reactive species in the brain are, as in the rest of the body, by-products of normal homeostatic functions such as protein degradation and energy production (Table 3).

## 8. Antioxidants and Neuropsychiatric Disorders

Despite these theoretical and practical difficulties, antioxidants have the potential to act as effective treatments for a variety of neuropsychiatric disorders. It has been previously established in patients with these neuropsychiatric disorders that there is an imbalance in the levels of antioxidants in the brain and blood plasma as well as some elements of mitochondrial dysfunction. For example, patients with AD were found to have decreased plasma levels of well-known antioxidants lycopene, vitamin A, vitamin C, and vitamin E [65]. Unfortunately, clinical trials directly treating the disorder with supplementation have not displayed positive results with some cases demonstrating a progressive decline in cognitive function in participants [66]. While these results are negative and do not support antioxidant therapy, a variety of factors such as the prooxidant effects of antioxidants and timing of administration can influence the outcome of the trial. Considering that reduced antioxidant enzyme activity, specifically superoxide dismutase, glutathione peroxidase, and glutathione reductase, and increased levels of 8-isoprostane were found in the CSF, plasma, and urine of patients with mild cognitive impairment (MCI) [65], a condition commonly seen in pre-AD patients, it is likely that the failure of antioxidant therapy in the treatment of AD can be ameliorated through earlier intervention. The concept of early intervention with antioxidant therapy still shows promise and should be investigated further in different contexts as the potential for an effective treatment across multiple neuropsychiatric disorders is high considering their common pathophysiological origins and mechanisms of progression.

## 9. Redox Modulations and Neuropsychiatric Disorders

Redox modulations play a major role in the development and progression of neuropsychiatric disorders [67]. Processes such as lipid peroxidation, protein and DNA oxidation, and mitochondrial dysfunction in the brain and periphery are indicative of neuropsychiatric disease, among other things. For example, mitochondrial dysfunction in PD [68] specifically complex I dysfunction [69] is linked to increased oxidative damage to the macromolecules and toxic products such as 4-hydroxynonenal (4HNE) found in PD. Moreover, 4HNE is correlated to damages to the 26/20S proteasome system [70] in PD.

In response to these toxic insults and enzymatic dysregulation, a broad-spectrum neuroprotective response is elicited that includes the increased expression of GSH peroxidase, succinic semialdehyde reductase, heme oxygenase-1, and NADPH dehydrogenase-1 enzymes. Considering that the degeneration of the SNpc is at least correlated with an increase in neuronal and astroglial NADPH dehydrogenase-1 expression, this constitutes a potential intervention point for therapeutics, including antioxidants. Whether artificial or natural inducers of endogenous antioxidant enzyme activity and the neuronal Nrf2 system could hypothetically lead to an amelioration of any neuropsychiatric pathology remains an open and challenging question for basic and translational research.

Furthermore, mitochondrial dysfunction leading to oxidative damage has long been linked with many neuropsychiatric disorders such as AD [71–75], bipolar disorder (BD) [76–81], major depressive disorder (MDD) [82–84], schizophrenia (SCZ) [85], Huntington's disease (HD) [86–88], and amyotrophic lateral sclerosis (ALS) [89–91]. In fact, there are common pathophysiological points between these neuropsychiatric disorders, emphasizing the possibility of common pharmacological intervention through synthetic or natural antioxidant compounds.

## 10. Future Directions

In light of the available evidence regarding antioxidants, it is clear that more studies are needed to explore their potential pharmacological properties. While there are many published and peer-reviewed studies regarding the mechanism of action and biological effects of antioxidants, there are few that seek to address the underlying issue of drug interactions, specifically with respect to medication prescribed for neuropsychiatric disorders. In order to supplement this growing body of research, clinical trials regarding the efficacy of antioxidants as potential stand-alone or adjunctive treatments need to be conducted. In addition, more studies are required to assess the long-term safety of antioxidants in healthy and nonhealthy individuals. From here, it becomes possible to closely examine the physicochemical properties of each antioxidant and use these as a basis for future drug development in the treatment of neuropsychiatric disorders and other various illnesses in accordance with previously established CNS drug characteristics [92].

## Figures and Tables

**Table 1:** Biological roles of reactive species.

Neurological	Cardiovascular	Immune response	Cell biology
Mediation of learning and memory Involved in the regulation of striatal dopamine release via glutamate	Regulation of cardiac contractility Regulation of vascular tone (e.g., penile erection) via NO production Signalling involving carotid bodies (monitor arterial oxygen levels)	Response to foreign pathogens (oxidative burst) Production of cytokines Wound repair	Embryogenesis Prevent overpopulation of cells and destroys malfunctioning cells Cellular differentiation

**Table 2:** Endogenous system of antioxidant enzymes.

Antioxidant enzyme	Cofactor/substrate	Reaction catalyzed	Location	Biochemical function
Copper-zinc-SOD (Cu, Zn-SOD, or SOD1) [93]	Copper and zinc^*∗*^	^∙^O_2_^−^ + ^∙^O_2_^−^ + 2H^+^ → H_2_O_2_	Cytosol, nucleus, mitochondria (intermembrane space)	Catalyzes the dismutation reaction of superoxide to H_2_O_2_ to decrease its reduction potential

Manganese SOD (MnSOD or SOD2) [93]	Manganese^*∗*^	^∙^O_2_^−^ + ^∙^O_2_^−^ + 2H^+^ → H_2_O_2_	Mitochondrial matrix	Same as above

Extracellular SOD (ecSOD or SOD3) [93]	Copper and zinc^*∗*^	^∙^O_2_^−^ + ^∙^O_2_^−^ + 2H^+^ → H_2_O_2_	Isoform secreted extracellularly	Same as above

Glutathione peroxidase (GPx) [93]	GSH^*∗∗*^ Selenium^*∗*^	(1) R-Se^−^H^+^ + ROOH/ONOO^−^ → ROH/ONO^−^ + R-SeOH **(**2) 2GSH + R-SeOH → GS-SG + R-Se^−^H^+^	Throughout the body	Reduce lipid hydroperoxides to alcohols and H_2_O_2_ to water

Glutathione-S-transferase (GST) [94]	GSH^*∗∗*^	GSH + RX → GSR + HX X = leaving group R = electrophilic group	Cytosol, mitochondria, peroxisome	Detoxification of xenobiotics

Glutathione reductase (GR) [94]	FAD^*∗*^ NADPH^*∗∗*^ GS-SG^*∗∗*^	GSSG + NADPH → 2GSH + NADP^+^	Cytosol, mitochondrial matrix	Maintenance of GSH levels

Catalase (CAT) [93]	Fe^2+^ and Fe^3+^^*∗*^	H_2_O_2_ → H_2_O + O_2_	Throughout the body; lowest in the brain	Reduces H_2_O_2_ to water and oxygen

Peroxiredoxins (Prx) [95]	Thioredoxin (Trx)^*∗∗*^	(1) Prx^red^ + H_2_O_2_ → Prx^ox^ + 2H_2_O (2) Prx^ox^ + Trx^red^ → Prx^red^ + Trx^ox^	Throughout the body (intracellular)	Reduces H_2_O_2 _to water Prx is reduced by Trx to be used in subsequent reactions

^*∗*^Cofactor; ^*∗∗*^substrate; SOD, superoxide dismutase.

**Table 3:** Various biological sources of reactive species in the brain.

	Source of reactive species in the brain	Function in the brain	General role in neuropathology
Organelle	Mitochondria [7]	Generates ATP	Defect or reduction in mitochondrial complex I/II/III/IV activity

Enzyme	Monoamine oxidases (MOA-A and MOA-B) [96]	Degrades neurotransmitters	Increased or decreased activity can lead to neurotransmitter imbalances as well as excess reactive species
	Nitric oxide synthase [97]	Synthesis of nitric oxide	Production of superoxide anion during normal NO^*∙*^ production
	Xanthine oxidase [98]	Catabolism of purines	Produces superoxide anions during normal metabolism
	Cytochrome P450 enzymes [99]	Drug metabolism Bioactivation of neurosteroids such as dehydroepiandrosterone (DHEA) Metabolism of retinoic acid (regulates gene expression) Cholesterol turnover in the brain	Reduced DHEA levels correlated with memory impairment Altered gene expression Reduced cholesterol turnover leading to accumulation in the brain

Metabolism	Arachidonic acid (AA) metabolism [97]	Maintains membrane fluidity Aids in the growth and repair of neurons Participates in activation of enzymes to store free fatty acids in the brain (prevents oxidative damage)	Elevated AA metabolism and/or overexpression of metabolizing enzymes Increased amounts of free fatty acids

## Abbreviations

ROS: Reactive oxygen species
RNS: Reactive nitrogen species
Nrf2: Nuclear factor (erythroid-derived 2)-like 2
Keap1: Kelch-like ECH-associated protein 1
PLC: Phospholipase C
PIP2: Phosphatidylinositol 4,5-bisphosphate
DAG: Diacylglycerol
IP3: Inositol triphosphate
PKC: Protein kinase C
Bach1: Transcription regulator protein BACH1
Maf: Transcription factor Maf
NQO1: NADH quinone oxidoreductase 1
HMOX-1: Heme oxygenase 1
GCL: Glutamate cysteine ligase
GST: Glutathione S-transferase
GSK3*β*: Glycogen synthase kinase 3 beta
GSH: Glutathione
DHLA: Dihydrolipoic acid.
